# Pretreatment lymphocyte-to-monocyte ratio as a prognostic factor and influence on dose-effect in fractionated stereotactic radiotherapy for oligometastatic brain metastases in non-small cell lung cancer patients

**DOI:** 10.3389/fonc.2023.1216852

**Published:** 2023-06-30

**Authors:** Tian Chen, Mengqiu Tang, Yang Zhou, Zhepei Wang, Shiwei Li, Hongcai Wang, Yangfang Lu, Jinguo Wang, Weiyu Shen

**Affiliations:** ^1^ Department of Radiation Oncology, Ningbo Medical Center Lihuili Hospital, Ningbo University, Ningbo, China; ^2^ Department of Ningbo Institute of Innovation for Combined Medicine and Engineering, Ningbo Medical Center Lihuili Hospital, Ningbo University, Ningbo, China; ^3^ Department of Neurosurgery, Ningbo First Hospital, Ningbo Hospital of Zhejiang University, Ningbo, China; ^4^ Department of Neurosurgery, Ningbo Medical Center Lihuili Hospital, Ningbo University, Ningbo, China; ^5^ Department of Thoracic Surgery, Ningbo Medical Center Lihuili Hospital, Ningbo University, Ningbo, China

**Keywords:** lymphocyte to monocyte ratio (LMR), biologically effective dose (BED), brain oligo-metastasis, fractionated stereotactic radiotherapy (FSRT), intracranial local control survival (i-LCS)

## Abstract

**Background:**

Studies on the prognostic factors for patients with brain oligo-metastasis treated with fractionated stereotactic radiotherapy (FSRT) usually focus on the size of metastatic tumor and radiation dose. Some inflammatory indicators have predictive value in non-small cell lung cancer (NSCLC) with brain metastasis receiving stereotactic radiotherapy. However, the prognostic value of inflammatory indicators in NSCLC patients with brain oligo-metastasis treated with FSRT, and their effect on radiotherapy dose is unknown.

**Methods:**

A total of 95 advanced NSCLC patients with brain oligo-metastasis who had undergone FSRT treatment at Ningbo Medical Center Lihuili Hospital between January 2015 and April 2022 were enrolled into the study. Neutrophil to lymphocyte ratio (NLR), platelet lymphocyte ratio (PLR), lymphocyte to monocyte ratio (LMR), tumor diameter and biologically effective dose (BED10) were analyzed using Chi-square test. Univariate and multivariate Cox regressions were used to identify predictors of survival.

**Results:**

Tumor diameter (< 2 cm), BED10 (≥ 48Gy) and LMR (≥ 4) were found to be independently associated with good intracranial local control survival (i-LCS) through multivariate analysis. The median i-LCS was longer in patients with 2 independent risk factors (tumor diameter ≥ 2 and LMR < 4) administered with BED10 > 53.6Gy compared with patients administered with BED10 ≤ 53.6Gy (20.7 months vs 12.0 months, *P* = 0.042). LMR ≥ 4 (*P* = 0.019) and positivity for driver gene mutations (*P* = 0.011) were independently associated with better overall survival (OS).

**Conclusions:**

LMR is an independent prognostic factor of i-LCS and OS in NSCLC patients with brain oligo-metastasis treated with FSRT. Patients with tumor diameter ≥ 2 and LMR < 4 should be treated with BED10 greater than 53.6Gy.

## Background

Brain metastases are the most common intracranial tumors in adult accounting for about 20-40 percent (1). Lung cancer is the most common primary malignant tumor that results in the brain metastases, with non-small cell lung cancer (NSCLC) accounting for more than 60 percent of the lung tumors (1, 2). The prognoses of patients with brain metastases arising from NSCLC varies greatly with the median survival time ranging from 6.9 to 46.8 months (3). Several high-technique models, such as diagnosis-specific graded prognostic assessment (DS-GPA), Graded Prognostic Assessment for Lung Cancer Using Molecular Markers (Lung-molGPA), score index for radiosurgery (SIR), and basic score for brain metastases (BSBM), are used to evaluate the prognosis of NSCLC patients (3–6), but the techniques are ineffective in evaluating the prognosis of NSCLC patients with brain oligo-metastasis treated with fractionated stereotactic radiotherapy (FSRT). FSRT has a higher local control rate and fewer side effects than stereotactic radiosurgery (SRS) therapy and has thus been widely used in the clinic. Compared with SRS therapy, FSRT showed a different biological effectiveness. For example, hypoxic tumor cells may survive after SRS, but FSRT, which is based on the principle of reoxidation, has better control rate in tumor (7), suggesting that the SIR model might not apply to the FSRT patients. There is need to identify prognostic factors for NSCLC patients with brain oligo-metastasis receiving FSRT therapy. Current studies on prognostic factors for oligo-metastasis patients treated with FSRT focus on the size of metastatic tumor and radiation dose. However, there is still no standard evaluation method for tumor size, radiation dose and fractionation scheme, with different studies suggesting different radiotherapy biologically effective dose (BED) (8–10). Therefore, there is need to develop and enhance predictive indexes indicating the efficacy and survival of NSCLC patients with brain oligo-metastasis receiving FSRT.

Since the discovery of the relationship between inflammation and cancer in 1863 by R Virchow (11), inflammatory indicators such as neutrophil-lymphocyte ratio (NLR), platelet to lymphocyte ratio (PLR), lymphocyte to monocyte ratio (LMR), regulatory T cells and peripheral memory CD4+ T cell, have been used in predicting efficacy and survival in different kinds of cancer (12–16). Inflammatory indicators have also been associated with prognosis of surgery, chemotherapy, targeted therapy, immunotherapy (17–20), and curative effect of radiotherapy (21, 22). In our previous study, we demonstrated that NLR, PLR and LMR constitute a simple and effective prediction index, in locally advanced esophageal cancer treated with surgery, and radiotherapy and in NSCLC treated with anti-vascular targeted therapy (23, 24). Several studies have also demonstrated the predictive value of some inflammatory indicators in NSCLC patients with brain metastasis receiving SRS therapy (25, 26). Although FSRT is similar to radiosurgery, the predictive value of inflammatory indicators in NSCLC patients with brain oligo-metastasis treated with FSRT and their effect on radiotherapy dose is unknown.

This study is a retrospective analysis of the association between inflammatory indicators (NLR, PLR and LMR) and the local control rate and survival of NSCLC patients with brain oligo-metastasis treated with FSRT, and their effect on radiotherapy dose.

## Materials and methods

### Patient selection

This was a retrospective study involving NSCLC patients with oligometastatic brain metastases who had been treated with FSRT at Ningbo Medical Center Lihuili Hospital between January 2015 and January 2022. The inclusion criteria was as follows: (i) pathological findings of metastatic or recurrent NSCLC; (ii) 3 or less brain metastases; (iii) FSRT used to treat brain metastases; (iv) availability of results for routine blood tests carried out two weeks prior to treatment. The exclusion criteria was as follows: (i) co-administration of FSRT and targeted drugs (osimertnib, almonertinib, furmonertinib, alectinib) during the stable disease stage; (ii) assessable focus was treated with FSRT previously; (iii) lack of relevant hematological data within 2 weeks prior to FSRT treatment; (iv) in an acute infection state when obtaining blood inflammation indicators; (v) absence of efficacy evaluation and follow-up information. In the end, 95 patients were enrolled into the study.

### Fractionated stereotactic radiotherapy technique

The FSRT treatment plan was based on the preference of the attending physician because the tumors were located near or within a critical structure. The head was first immobilized with an aquaplast, and then a computed tomography (CT) scan with intravenous contrast was acquired to plan radiotherapy. Fusing magnetic resonance (MR) T1-weighted imaging with CT images within two weeks of treatment planning. In CT and MR images, the gross tumor volume (GTV) was defined as the contrast medium-enhancing tumor, the clinical target volume (CTV) represented the GTV, while the planning target volume (PTV) was considered as CTV plus a 2-4mm margin. A single split dose of FSRT was set from 3.5-7Gy. Approximately 90% of the maximum dose was applied to the peripheral area, and 95% of the PTV was covered by the peripheral dose. Radiation therapy was administered 5 times a week. We evaluated the dose response of various FSRT fractionation schedules according to a biologically effective dose using an alpha/beta ratio of 10 (BED10) as a measure of the biological effectiveness of the treatment.

### Analysis of laboratory parameters

The following hematology indexes were evaluated up to 2 weeks prior to FSRT: neutrophil count (× 10^9^/L), platelet count (× 10^9^/L), lymphocyte count (× 10^9^/L) and monocytes count (× 10^9^/L). NLR was defined as the neutrophil count divided by the lymphocyte count. Similarly, PLR was the ratio of the platelet count to the lymphocyte count, LMR was the ratio of the lymphocyte count to the monocytes count. The cutoff values were defined as 5, 180 and 4 for NLR, PLR, and LMR, respectively (25, 27–29). For tumor diameter, the cut off value was 2cm (median tumor diameter), and for BED 10, the cut off value was 48 Gy.

### Outcome evaluation and statistics

Taking brain enhanced MR re-examination after FSRT in 1-2 months, subsequently checking per 2-3 months, checking enhanced MR immediately at the appearance of intracranial hypertension or neuropsychiatric symptoms. Intracranial local control (i-LC) was defined as no significant increase in the size tumor lesion treated with FSRT on follow-up MR. Intracranial local control survival (i-LCS) was the primary end point of assessment. It was defined as the time from the start of radiation therapy to the time enlargement of the tumor treated with FSRT was observed. Overall survival (OS) was calculated from the date of initiation of FSRT to the time of death from any reason or last time of follow up. OS was the secondary end point of assessment.

The statistical analyses were performed using a social science statistical software package, version 26.0 (SPSS Inc., Chicago, IL, US). Chi-squared tests were used to analyze categorical variables. A Kaplan-Meier survival curve was plotted and compared with a log-rank-test curve. Factors for survival were identified using univariate and multivariate Cox regression analyses. Statistical significance was deemed to be a *P*-value < 0.05.

## Results

### Patient characteristics and curative effect

The median age of the study participants was 63 years (range from 37 to 79 years).Adenocarcinoma was the most common type of cancer (n = 79, 83.2%), with 13 patients having squamous cell carcinomas, 2 patients having poorly differentiated carcinoma, and 1 patient having large cell carcinoma. Only 11 patients had karnofsky performance status (KPS) scores less than 80, while the rest had scores greater than or equal to 80. The maximum diameter of intracranial metastases ranged from 0.6 to 6.4cm, with a median diameter of 2.0cm. A total of 38 patients (40.0%) had confirmed EGFR mutations, 3 patients (5.1%) had ALK rearrangement and one patient had MET-14 jumping mutation. Most of the patients had no history of brain radiotherapy (n = 84, 88.4%). The median BED10 was 53.6Gy (range from 37.5 to 85.1 Gy) and the number of splits was 5-18. The detailed information of patient characteristics and baseline data are shown in **Table 1**.

**Table 1:** Basic characteristics of non-small cell lung cancer patients with oligometastatic brain metastases.

Characteristics	Patients (%)
Age (years)	
Median	63 years
Range	37-79
< 65	55 (57.9%)
≥ 65	40 (42.1%)
Gender	
Male	45 (47.4%)
Female	50 (52.6%)
Karnofsky performance status (%)	
≥ 80	84 (88.4%)
< 80	11 (11.6%)
Histologic subtype	
Adenocarcinoma	79 (83.2%)
Squamous cell carcinoma	13 (13.7%)
Other	3 (3.1%)
Driver gene mutation	
Positive	42 (44.2%)
Negative or unknown	53 (55.8%)
Number of brain metastases	
1	71 (74.7%)
2-3	24 (25.3%)
Localization of brain metastases	
Supratentorial	81 (85.3%)
Infratentorial	14 (14.7%)
CNS treatment before FSRT	
None	84 (88.4%)
WBRT	4 (4.2%)
FSRT not in this location of metastases	7 (7.4%)
Extracranial metastases	
Yes	74 (77.9%)
No	21 (22.1%)
Maximum diameter of brain metastases (cm)	
Median	2.0
Range	0.6-6.4
>2	44 (46.3%)
≥2	51 (53.7%)
BED10 of FSRT (Gy)	
Median	53.6
Range	37.5-85.1
>48	16 (16.8%)
48-53.6	50 (52.6%)
<53.6	29 (30.6%)
NLR	
Median	2.75
Range	0.78-9.25
>5	70 (73.7%)
≥5	25 (26.3%)
PLR	
Median	152.00
Range	52.38-698.00
>180	65 (68.4%)
≥180	30 (31.6%)
LMR	
Median	2.57
Range	0.83-16.00
>4	67 (70.5%)
≥4	28 (29.5%)

FSRT fractionated stereotactic radiotherapy, WBRT whole brain radiation therapy, BED10 biologically effective dose (α/β = 10), NLR neutrophil to lymphocyte ratio, PLR platelet lymphocyte ratio, LMR lymphocyte to monocyte ratio.

The follow-up time ranged from 3.0 to 37.7 months, with a median of 20.6 months. The local control rates at 6 and 12 months were 82.9% and 66.5%, respectively. The median i-LCS and OS were 15.8 months and 19.4 months, respectively. (**Figure S1A, B**).

### Factors associated with intracranial local control survival and their effect on radiotherapy dose

**Figure 1:**
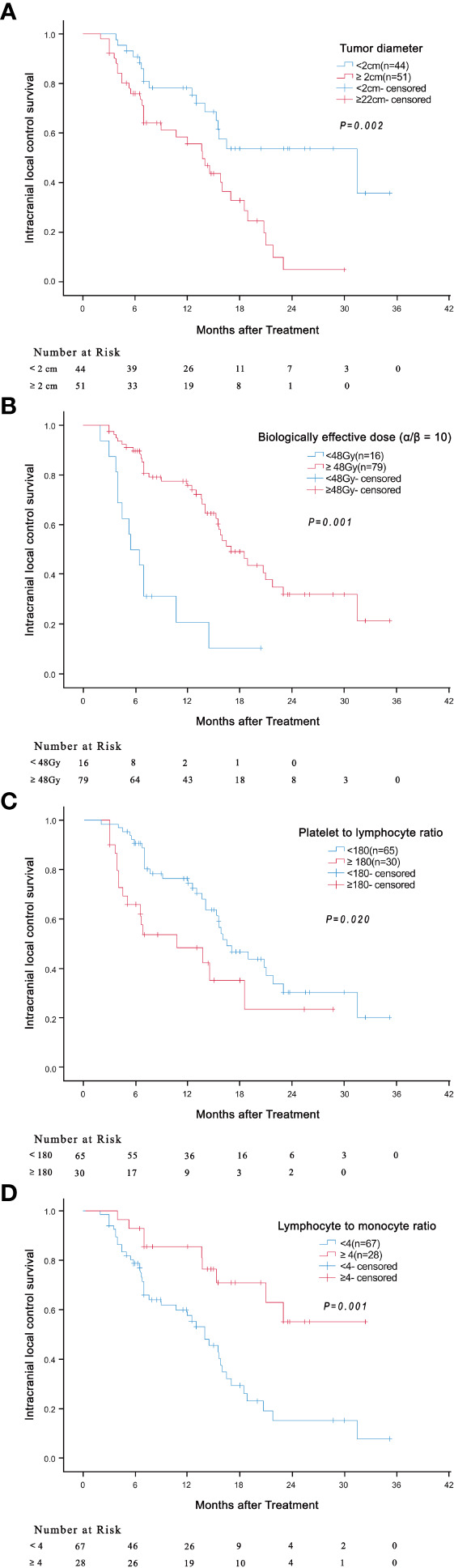
Association of tumor diameter (≥ 2cm versus < 2cm) (**(A)** *P* = 0.002), BED10 (≥ 48Gy versus < 48Gy) (**(B)** *P* = 0.001), PLR (≥ 180 versus < 180) (**(C)** *P* = 0.020) and LMR (≥ 4 versus < 4) (**(D)** *P* = 0.001) with intracranial local control survival.

Univariate analyses found that tumor diameter, BED10, PLR and LMR were significant risk factors for i-LCS. Other factors including age, gender, number of brain metastases and presence of extracranial metastasis at diagnosis were not associated with i-LCS in univariate analyses (**Table 2**). Median i-LCS was significantly shorter in patients with tumor diameter ≥ 2 cm, compared to patients with smaller tumors (13.7 months vs. 31.5 months, HR: 2.595, 95% CI: 1.417-4.750, P = 0.002) (**Figure 1A**). Median i-LCS was significantly longer in patients with BED10 ≥ 48Gy, compared to patients with less BED10 (17.0 months vs 5.5 months, HR: 0.241, 95% CI: 0.124-0.468, *P* = 0.001) (**Figure 1B**). Median i-LCS was significantly longer among patients with PLR ≥ 180, compared to patients with lower values (10.7 months vs 16.5 months, HR: 2.023, 95% CI: 1.116-3.669, *P* = 0.020) (**Figure 1C**). Median i-LCS was significantly longer in patients with LMR ≥ 4, compared to patients with lower values (not reached vs 14.0 months, HR: 0.306, 95% CI: 0.147-0.636, *P* = 0.001) (**Figure 1D**). The results of multivariate analysis demonstrated that tumor diameter (< 2 cm), BED10 (≥ 48Gy) and LMR (≥ 4) were independently associated with good i-LCS (**Table 3**).

**Table 2:** Univariate analysis of factors associated with intracranial local control survival and overall survival.

Prognostic factors	Intracranial local control survival			Overall survival		
	HR	95% CI	*P*-value	HR	95% CI	*P*-value
Age (years)						
< 65	1			1		
≥ 65	0.948	0.536-1.677	0.855	0.891	0.497-1.596	0.698
Gender						
Female	1			1		
Male	0.981	0.558-1.724	0.946	1.185	0.673-2.086	0.557
KPS (%)						
< 80	1			1		
≥ 80	0.689	0.321-1.477	0.339	1.260	0.498-3.188	0.626
Histologic subtype						
Adenocarcinoma	1			1		
Others	1.627	0.785-3.370	0.191	1.811	0.920-3.564	0.086
Driver gene mutation						
Negative or unknown	1			1		
Positive	0.675	0.384-1.188	0.173	0.417	0.226-0.769	0.005
Number of brain metastases						
1	1			1		
2-3	0.851	0.457-1.584	0.611	0.887	0.475-1.657	0.707
Localization of brain metastases						
Supratentorial	1			1		
Infratentorial	1.127	0.501-2.535	0.773	0.447	0.138-1.447	0.179
CNS treatment before FSRT						
None	1			1		
RT	0.636	0.251-1.611	0.340	0.457	0.141-1.478	0.191
Extracranial metastases						
No	1			1		
Yes	0.876	0.464-1.654	0.683	1.073	0.544-2.117	0.840
Maximum diameter of brain metastases (cm)						
< 2	1			1		
≥ 2	2.595	1.417-4.750	0.002	1.265	0.719-2.225	0.414
BED10 of FSRT (Gy)						
< 48	1			1		
≥ 48	0.241	0.124-0.468	0.001	0.903	0.421-1.937	0.793
NLR						
< 5	1			1		
≥ 5	1.104	0.592-2.056	0.756	1.261	0.678-2.343	0.464
PLR						
< 180	1			1		
≥ 180	2.023	1.116-3.669	0.020	1.856	1.024-3.366	0.042
LMR						
< 4	1			1		
≥ 4	0.306	0.147-0.636	0.001	0.408	0.197-0.843	0.015

HR hazard ratio, CI confidence interval, KPS karnofsky performance status, CNS central nervous system, FSRT fractionated stereotactic radiotherapy, RT radiotherapy, BED10 biologically effective dose (α/β = 10), NLR neutrophil to lymphocyte ratio, PLR platelet to lymphocyte ratio, LMR lymphocyte to monocyte ratio

**Table 3:** Multivariate analysis of factors associated with intracranial local control survival and overall survival.

Prognostic factors	Intracranial local control survival			Overall survival		
	HR	95% CI	*P*-value	HR	95% CI	*P*-value
Maximum diameter of brain metastases (< 2cm *vs* ≥ 2cm)	1.945	1.041-3.636	0.037	/	/	/
BED10 of FSRT (< 48Gy *vs* ≥48Gy)	0.268	0.130-0.553	0.001	/	/	/
PLR (< 180 *vs* ≥ 180)	1.369	0.716-2.618	0.342	1.476	0.800-2.723	0.212
LMR (< 4 *vs* ≥ 4)	0.365	0.171-0.776	0.009	0.414	0.198-0.864	0.019
Driver gene mutation (negative or unknown *vs* positive)	/	/	/	0.442	0.236-0.828	0.011

BED10 biologically effective dose (α/β = 10), FSRT fractionated stereotactic radiotherapy, PLR platelet to lymphocyte ratio, LMR lymphocyte to monocyte ratio

**Figure 2:**
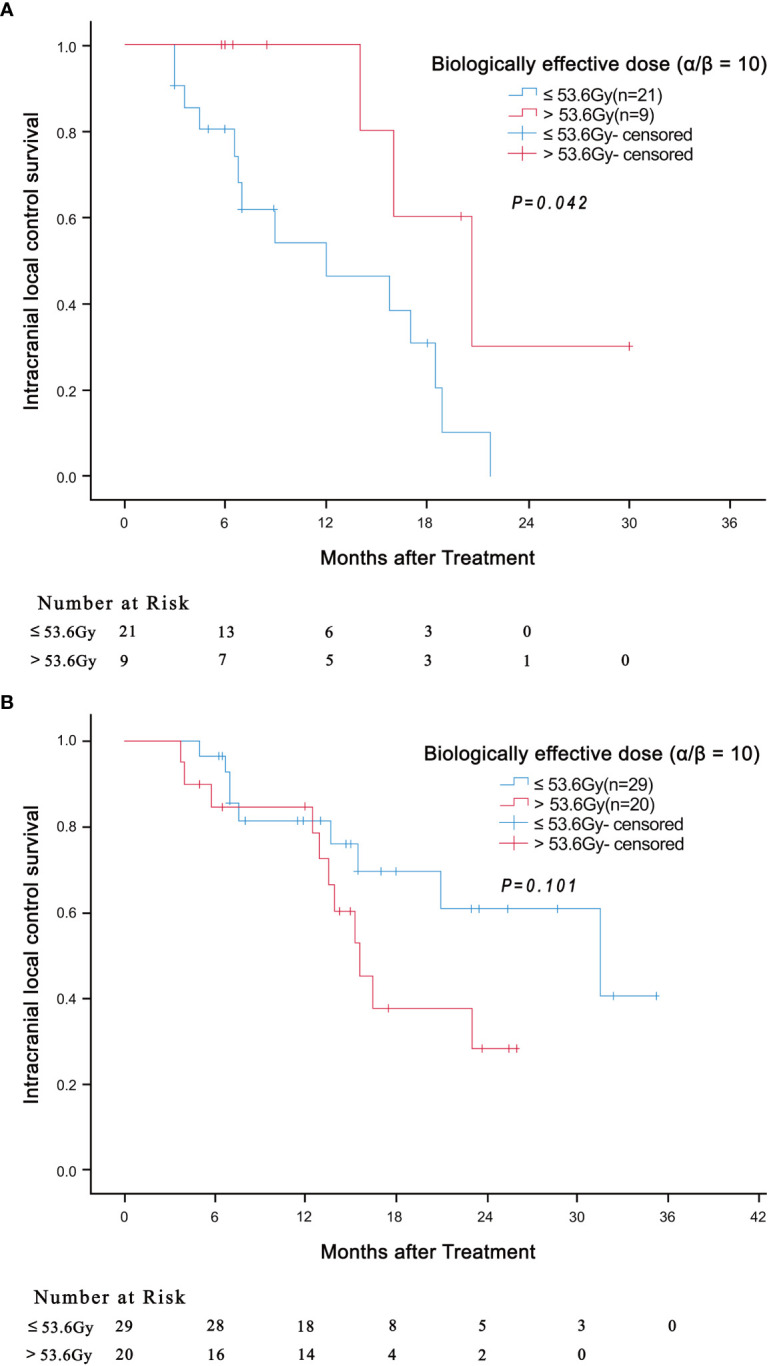
Association of BED10 (> 53.6Gy versus ≤ 53.6Gy) with intracranial local control survival in patients with 2 independent risk factors (tumor diameter ≥ 2 and LMR < 4) (**(A)** *P* = 0.042) and in patients with only 1 independent risk factor (**(B)** *P* = 0.101).

We then evaluated the effect of the independent prognostic factors on radiotherapy among the patients with BED10 ≥ 48Gy. In total, 49 patients had BED10 ≥ 48Gy, with the median BED10 being 53.6Gy. Surprisingly, in patients with 2 independent risk factors (tumor diameter ≥ 2 and LMR < 4), the i-LCS was longer in patients with BED10 greater than 53.6Gy, compared to patients with BED10 less than 53.6Gy (20.7 months vs 12.0 months, HR: 0.290, 95% CI: 0.082-1.030, *P* = 0.042) (**Figure 2A**). There was no significant difference in i-LCS among patients with different BED10 values and with only 1 independent risk factor (*P* = 0.101, **Figure 2B**)

### Factors associated with overall survival

**Figure 3:**
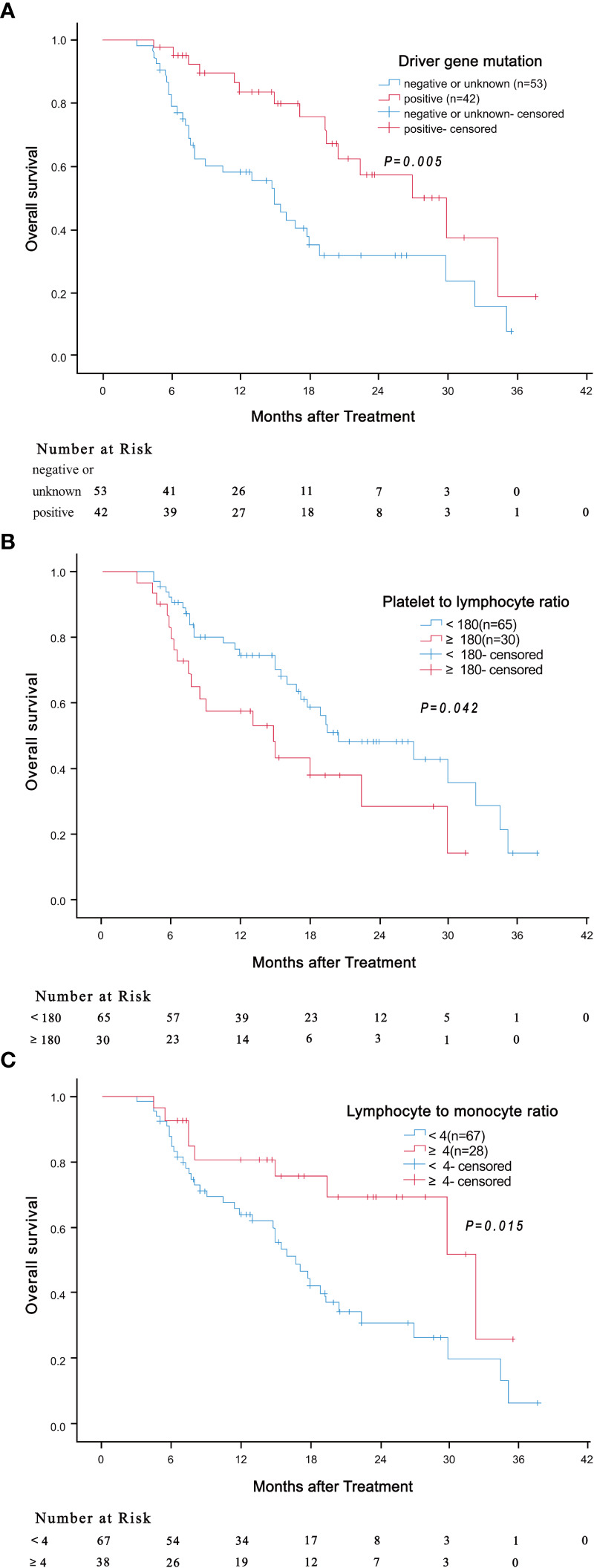
Association of driver gene mutation (**A**, *P* = 0.005), PLR (**B**, *P* = 0.042) and LMR (**C**, *P* = 0.015) with overall survival.

Results of univariate analysis revealed that driver gene mutations, PLR< 180 and LMR ≥ 4 were associated with better OS, but not BED10 (**Table 2**). Patients positive for driver gene mutations had longer median OS than patients negative for driver gene mutations or with unknown mutations (30.0 months vs 15.0 months, HR: 0.417, 95% CI: 0.226-0.769, *P* = 0.005) (**Figure 3A**). The median OS of patients with PLR <180 was significantly shorter compared to patients with higher values (14.8 months vs 20.5 months, HR: 1.856, 95% CI: 1.024-3.366, *P* = 0.042) (**Figure 3B**). The median OS of patients with LMR ≥ 4 was significantly longer compared to patients with lower values (32.4 months vs 16.8 months, HR: 0.408, 95% CI: 0.197-0.843, *P* = 0.015) (**Figure 3C**). Multivariate analysis demonstrated that LMR ≥ 4 and presence of driver gene mutations were independently associated with better OS (**Table 3**).

## Discussion

To our knowledge, this is the first study to evaluate the predictive value of inflammatory indicators in NSCLC patients with brain oligo-metastasis treated with FSRT. Our results showed that, in addition to tumor diameter and BED 10, LMR (an inflammatory indicator), was also an independent prognostic factor for i-LCS. Tumor diameter and BED10 had previously been identified as prognostic factors for i-LCS (9, 30, 31), but our study is the first to identify LMR value as an independent prognostic factor. Anna Cho et al. (25) reported that NLR, PLR and LMR were independent prognostic factors of overall survival in NSCLC patients with brain metastases after Gamma Knife Radiosurgery. However, Aijie Li et al. (32) proposed that LMR was the only independent prognostic factor of overall survival in NSCLC patients with brain metastases. The differences in these finding could be due to differences in the study population. The participants in our study were all NSCLC patients with oligometastatic brain metastases who had undergone FSRT treatment. We found that LMR as the only inflammatory indicator that acted as an independent prognostic factor of overall survival, although PLR was identified as prognostic factor during univariate analysis but was negative in multivariable analysis. Therefore LMR ≥ 4 is an excellent independent prognostic factor for i-LCS and overall survival in NSCLC patients with oligometastatic brain metastases treated with FSRT. We also found that positivity for driver gene mutations was an independent prognostic indicator of overall survival, which was consistent with the findings of Aijie Li et al. (32). The reason why driver gene mutations were independent prognostic indicators of overall survival but not i-LCS may be attributed to the overall poor extracranial control rate in patients negative for driver gene mutations, which leads to the death from extracranial lesions.

It is generally acknowledged that the cut-off value of LMR is 4 (25, 26), which was confirmed in our study. There is no consensus on the cut-off value of tumor diameter. Some articles indicate that the cut-off value of tumor diameter is 1cm in brain metastases receiving radiosurgery (33), and 2cm or 3cm in brain metastases receiving FSRT (34, 35). The i-LCS was significantly better in patients with lower cut-off value. Our results showed the cut-off value was 2cm demarcated by the median value. The median i-LCS was just 13.7 months in patients with tumors above 2cm but was as long as 31.5 months in patients with tumors below 2 treated with FSRT. The effective radiation dose for clinical application is still controversial. It is generally agreed that the radiation dose cut-off value for SRS treatment in brain metastases is 18Gy, with local control deteriorating significantly in patients receiving the dose below 18Gy (36). The use of BED is determining the curative effect of SRS is debatable (37), but is usually applied in determining the curative effect of FSRT treatment. Several divide-up radiotherapy plans have been suggested (8–10). One review summarized and compared the curative effect of different BED values, and concluded that BED12 values greater than 40Gy (which equals to BED10 greater than 48Gy) achieved a higher local control rate (38). Another study showed the 1 year local control rate was 100% for BED10 greater than 48Gy but was only 33% for BED10 less that 48Gy in treating postoperative metastasis tumor bed using FSRT (39). Thus it is generally believed the cut-off value of BED10 is 48Gy. In our study, we found that the median i-LCS of patients who received BED10 greater than 48Gy was significantly longer than for patient who received less BED10 (17.0 months vs 5.5 months). Results from multivariate analysis indicated the BED10 was an independent prognostic factor of i-LCS. Samuel R et al. (34) assessed if enhancement of BED value improved i-LCS, and found that enhancing BED10 value did not improve i-LCS in patients with a tumor diameter of more than 3cm. However, we found that administration of BED 10 greater than 53.6Gy improved the i-LCS of patients with two independent risk factors (tumor diameter ≥ 2 and LMR < 4), but had no benefit in the patients with 1 or no independent risk factor. The difference in findings may be because the study by Samuel R et al. enrolled patients with only 1 independent risk factor (tumor diameter ≥3cm) and did not screen the patients for obstinate resistance to radiotherapy. Unlike their results, this study analyzed the LMR value below 4 as the other independent risk factor. Thus got benefit by improving BED value in patients simultaneously possessed two independent risk factors (tumor diameter ≥ 2 and LMR < 4) equivalent to possessing obstinate resistance to radiotherapy.

Neutrophils inhibit immune functions and induce resistance to chemoradiotherapy by secreting cytokines and chemokines (13, 40, 41). Platelets are a critical source of cytokines, such as transforming growth factor-β, platelet-derived growth factor, and vascular endothelial growth factor (VEGF), which induce angiogenesis and cell invasion (42, 43). Moreover, lymphocytes can produce several cytokines, including IFN-γ and perforin, to prevent tumor development and induce apoptosis in cancer cells (36). Monocytes are innate immune cells that play important roles in tumor progression, invasion and metastasis and can be grouped into macrophages and myeloid-derived suppressor cells (44, 45). These findings from literature suggest that NLR, PLR and LMR have potential roles as prognostic factors in tumor development and treatment. In our study, we found that LMR but not NLR was a prognostic factor in the NSCLC patients with oligometastatic brain metastases receiving FSRT treatment. The difference between the two factors could be due to the effect of glucocorticoids. Glucocorticoids are usually administered to reduce the intracranial pressure before radiation therapy once the diagnosis of brain metastasis has been confirmed. The use of glucocorticoids can affect neutrophil counts which affects NLR value resulting in a negative result. It is also possible that NLR has no prognostic value in patients receiving FSRT treatment.

There are some limitations in our study. First, this was a retrospective study involving a small sample from a single center, which may have caused analytical bias. Second, the lack of follow-up data for different treatments before and after the radiotherapy may have influenced the analysis. Third, adverse reactions such as acute cerebral edema and radionecrosis are difficult to detect and were not reported during follow-up, thus adverse reactions analysis could not be carried out. Fourthly, selection bias may have been present despite the strict inclusion criteria, and thus the findings need to be validated in future prospective studies. Fifth, a part of patients from our study had been pronounced dead because of the extracranial lesions before the intracranial lesions, and this point was linked to the end of intracranial follow-up which resulted in attrition bias. Therefore, there is need for multi-center prospective randomized clinical trials with large sample size to validate the prognostic value of LMR in NSCLC patients with oligometastatic brain metastases treated with FSRT and its effect on radiotherapy dose.

## Conclusions

LMR is a prognostic factor for i-LCS and OS in NSCLC patients with oligometastatic brain metastases treated with FSRT. The basic dose for BED10 was greater that 48Gy, but should be increased to greater than 53.6Gy in patients with tumor diameter ≥ 2 and LMR < 4.

## Data availability statement

The raw data supporting the conclusions of this article will be made available by the authors, without undue reservation.

## Ethics statement

The studies involving human participants were reviewed and approved by Ningbo Medical Center Lihuili Hospital ethics committee. Written informed consent for participation was not required for this study in accordance with the national legislation and the institutional requirements. Written informed consent was not obtained from the individual(s) for the publication of any potentially identifiable images or data included in this article.

## Author contributions

TC and WS contributed to the conception and design of the study. TC and MT wrote the article together. SL, YL, and JW contributed to the acquisition and analysis of the data. YZ, ZW, and HW participated in revising of the article. All authors contributed to the article and approved the submitted version.

## Supplementary material

Supplementary Figure 1 Curves of intracranial local control survival **(A)** and overall survival **(B)**.
