# Supplementary figures and images for: Pretreatment lymphocyte-to-monocyte ratio as a prognostic factor and influence on dose-effect in fractionated stereotactic radiotherapy for oligometastatic brain metastases in non-small cell lung cancer patients
Source: Front Oncol. 2023 Jun 30;13:1216852. doi: 10.3389/fonc.2023.1216852 (PMC10348423; doi:10.3389/fonc.2023.1216852)

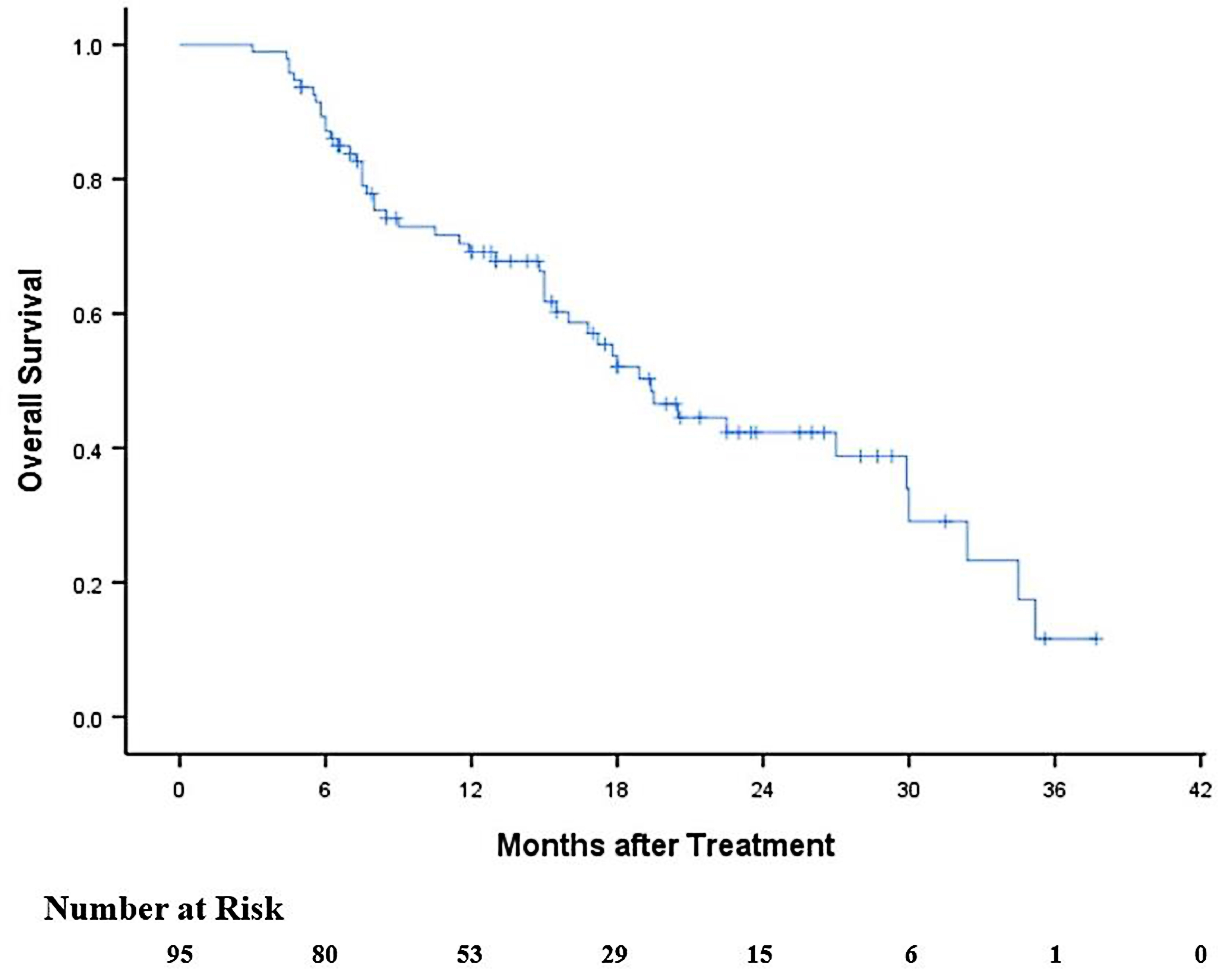

Supplement: Supplementary file 1 [file Image_2.jpeg]

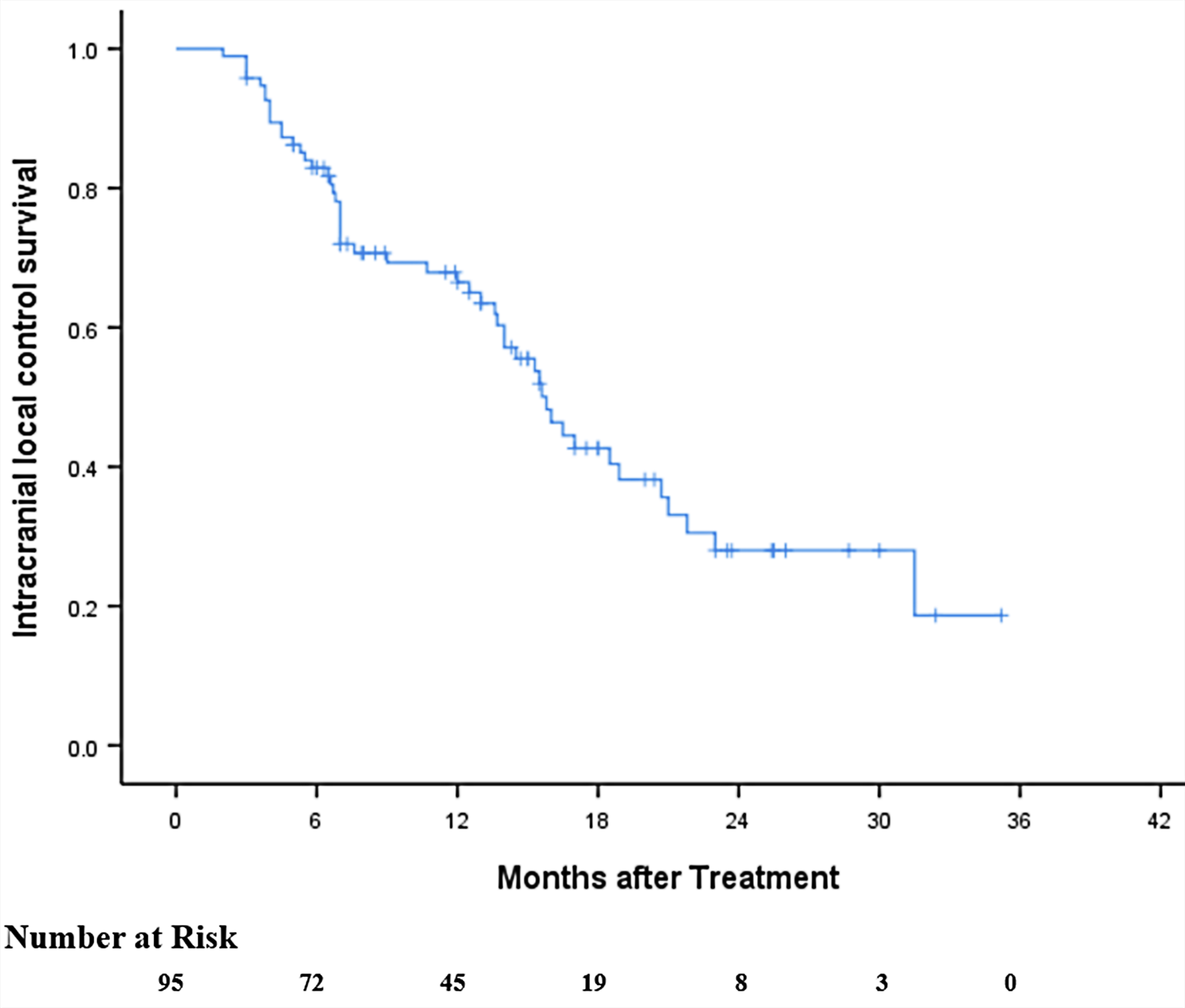

Supplement: Supplementary Figure 1 — Curves of intracranial local control survival (A) and overall survival (B). [file Image_1.jpeg]
